# Clinical diagnostic accuracy of acute colonic diverticulitis in patients admitted with acute abdominal pain, a receiver operating characteristic curve analysis

**DOI:** 10.1007/s00384-016-2644-0

**Published:** 2016-09-09

**Authors:** A. Jamal Talabani, B. H. Endreseth, S. Lydersen, T.-H. Edna

**Affiliations:** 1 Department of Surgery, Levanger Hospital, Nord-Trøndelag Hospital Trust, N-7602 Levanger, Norway; 2 Unit for Applied Clinical Research, Department of Cancer Research and Molecular Medicine, Faculty of Medicine, Norwegian University of Science and Technology, Trondheim, Norway; 3 Clinic of Surgery, St Olavs Hospital, University of Trondheim, Trondheim, Norway; 4 Department of Cancer Research and Molecular Medicine, Faculty of Medicine, Norwegian University of Science and Technology, Trondheim, Norway; 5 Regional Centre for Child and Youth Mental Health and Child Welfare – Central Norway, Faculty of Medicine, Norwegian University of Science and Technology, Trondheim, Norway

**Keywords:** Acute colonic diverticulitis, Clinical diagnostic accuracy, Receiver operating characteristic curve, C-reactive protein, White blood cell count, Temperature

## Abstract

**Purpose:**

The study investigated the capability of clinical findings, temperature, C-reactive protein (CRP), and white blood cell (WBC) count to discern patients with acute colonic diverticulitis from all other patients admitted with acute abdominal pain.

**Methods:**

The probability of acute diverticulitis was assessed by the examining doctor, using a scale from 0 (zero probability) to 10 (100 % probability). Receiver operating characteristic (ROC) curves were used to assess the clinical diagnostic accuracy of acute colonic diverticulitis in patients admitted with acute abdominal pain.

**Results:**

Of 833 patients admitted with acute abdominal pain, 95 had acute colonic diverticulitis. ROC curve analysis gave an area under the ROC curve (AUC) of 0.95 (CI 0.92 to 0.97) for ages <65 years, AUC = 0.86 (CI 0.78 to 0.93) in older patients. Separate analysis showed an AUC = 0.83 (CI 0.80 to 0.86) of CRP alone. White blood cell count and temperature were almost useless to discriminate acute colonic diverticulitis from other types of acute abdominal pain, AUC = 0.59 (CI 0.53 to 0.65) for white blood cell count and AUC = 0.57 (0.50 to 0.63) for temperature, respectively.

**Conclusion:**

This prospective study demonstrates that standard clinical evaluation by non-specialist doctors based on history, physical examination, and initial blood tests on admission provides a high degree of diagnostic precision in patients with acute colonic diverticulitis.

## Introduction

As the incidence and admission rates of acute colonic diverticulitis are rising in the Western world, the burden on healthcare systems increases [1–4]. The recurrence rate is high, ranging from 6 to 25 % in the subsequent years after the first episode [2, 5], causing repeated consultation and treatment in the primary healthcare and potential hospital readmission. Patients with uncomplicated acute diverticulitis do not necessarily require hospital admission and may be treated on an out-patient basis. Thus, differentiated treatment guidelines of evidence-based clinical care pathways would be effective for the patient and cost saving for the healthcare system [6, 7].

During recent decades, a significant change in both diagnostics and treatment of acute colonic diverticulitis has evolved. As a supplement to clinical evaluation, computed tomography (CT) scans verify and determine stage of acute colonic diverticulitis, improving the decision-making in non-operative and operative management [8, 9]. CT scan is a cost-effective way to diagnose acute abdominal conditions; however, radiation exposure is a concern [10]. Patients with uncomplicated colonic diverticulitis are treated conservatively, and the role of antibiotics is disputable [11, 12]. Radiological drainage of localized abscesses has been introduced [13], and surgical intervention has changed from major open procedures to more conservative laparoscopic procedures in selected cases [14].

The rate of complicated acute colonic diverticulitis is highest during the primary admission, and the majority of readmitted patients have uncomplicated disease [2, 15]. A repeated CT scan may be omitted in patients with clinical recurrent disease and a previously documented history of diverticulitis, especially with C-reactive protein (CRP) <50 mg/mL [16]. A clinical diagnosis of uncomplicated diverticulitis, which comprises the majority of these patients [8, 12], remains of major importance in the management of this condition in an out-patient setting [17].

The main aim of the present prospective study was to assess the accuracy of clinical diagnosis in acute colonic diverticulitis, based on history, physical examination, and initial blood tests on admission to hospital in patients with acute abdominal pain, using a receiver operating characteristic curve (ROC) analysis. The secondary aim was to examine the diagnostic accuracy of temperature, CRP, and white blood cell (WBC) count alone in the diagnosis of acute colonic diverticulitis in patients admitted with acute abdominal pain.

## Methods

Between November 2011 and March 2014, all patients older than 18 years, who were admitted to the Department of Surgery at Levanger Hospital with acute abdominal pain with a duration of less than 1 week, were invited to participate in the study. The hospital, located in Mid-Norway, is a first-line hospital serving the population of ten municipalities in North-Trondelag County, which had 94,174 inhabitants in 2012.

After admission, 833 patients gave written consent to participate and were included in the study; 477 (57 %) were women, and 537 (64 %) were below 65 years of age.

Based on anamnestic history, primary clinical examination, temperature and the results of initial blood tests (CRP and WBC), and before the results of any radiological examinations were available; the non-specialist doctors (usually pre-registrar house officers) in the emergency department scored the probability of acute colonic diverticulitis for the particular patient. A specific form, based on a categorized score from 0 (zero probability) to 10 (100 % probability) was used. In all, 107 different non-specialist doctors were involved in the scoring, 20 of these examined 50 % of the included patients.

The final diagnosis in all patients included in the study was based on the diagnosis at discharge and supplementary radiologic or endoscopic examinations as part of an ambulatory follow-up. Acute non-specific abdominal pain (NSAP) was defined as acute abdominal or pelvic pain without any obvious pathology if routine investigations, which included imaging and blood tests, did not reveal pathology and if the patient responded to non-specific treatment [18].

Acute colonic diverticulitis was confirmed by CT scan before discharge in 83 of 95 patients. Five patients with recurrent acute colonic diverticulitis had a recent CT verifying acute colonic diverticulitis, and five had their diagnosis confirmed by an ambulant CT scan or colonoscopy after discharge. Discharge diagnosis based on clinical examination and laboratory tests only occurred twice; the patients were aged 77 and 81 years.

In the study period, an unknown number of patients admitted with acute abdominal pain refused inclusion. Review of data from the hospital’s patient administrative system and patient records revealed a total of 168 patients admitted with acute colonic diverticulitis as discharge diagnosis in the relevant time period; of these, 95 (57 %) were included in the study. There were no statistical differences in gender, age, subtype of acute colonic diverticulitis, or length of hospital stay between patients included and those not included in the study, as shown in Table 1.

**Table 1:** Characteristics of patients with acute colonic diverticulitis included in the study compared with those not included

	Patients included in the study (*n* = 95)	Patients not included in the study (*n* = 73)	Total (*n* = 168)	*p* value
Male	34 (36)	21 (29)	55 (33)	0.34^b^
Age (years)^a^	61 (28–90)	66 (28–92)	62 (28–92)	0.29^c^
Hospital stay (days)^a^	2 (0–19)	2 (1–59)	2 (0–59)	0.045^c^
Uncomplicated diverticulitis	80 (84)	60 (82)	140 (83)	0.54^d^
Hinchey 1–2	10 (11)	5 (7)	15 (9)	0.54^d^
Bowel obstruction (acute operation)	0	2 (3)	2 (1)	0.54^d^
Hinchey 3	2 (2)	5 (7)	7 (4)	0.54^d^
Hinchey 4	3 (3)	1 (1)	4 (2)	0.54^d^

Values in parentheses are percentages, unless indicated otherwise

^a^Values are median (range)

^b^Z-pooled exact test

^c^Wilcoxon test

^d^Cochran-Armitage Trend test

### Statistical analysis

Proportions were compared using the unconditional z-pooled exact test. The medians of two samples were compared using the Wilcoxon test. The diagnostic accuracy of the clinical score was tested using ROC curves, including the area under the curve (AUC). An AUC value of 0.5 shows no predictive ability for the test in question, whereas a value of 1.0 indicates perfect discrimination [19]. Strength of discrimination has been classified as the following: AUC = 0.5 denotes no discrimination, AUC = 0.7–0.79 corresponds to acceptable strength of discrimination, AUC = 0.8–0.89 corresponds to excellent discrimination strength, and AUC = 0.9–1.0 corresponds to outstanding discrimination strength [20].

Two basic measures (sensitivity and specificity) of diagnostic accuracy were used [21] calculated from ROC curve analysis and expressed in percentage.

Two-sided *p* values <0.05 were considered significant. Ninety-five percent confidence intervals (CI) were reported where relevant. Medians are reported with range (minimum to maximum) where relevant.

The analyses were performed using SPSS 22 (SPSS Inc., Chicago, IL, USA), STATA 13 (Stata Corp LP, College Station, TX, USA), and StatXact 9 (Cytel Inc., Cambridge, MA, USA).

### Ethics

The Regional Committee for Medical and Health Research Ethics (REC) gave permission for the study (2011/1782/REK midt). Only patients who gave written consent were included in the study.

## Results

The study included 833 patients admitted with acute abdominal pain. The final diagnosis in relation to age is shown in Table 2. Overall, NSAP was the most frequent diagnosis, in 23 % of the patients, and acute colonic diverticulitis was the third most common diagnosis, in 11 % of the patients. Among patients younger than 65 years, NSAP was the most frequent diagnosis, while acute colonic diverticulitis was fourth in frequency. Among patients older than 65 years, acute biliary disease was the most frequent diagnosis, and acute colonic diverticulitis was third in frequency.

**Table 2:** Final diagnosis in patients admitted with acute abdominal pain, in relation to age

Final diagnosis	< 65 years	65 + years	Total
Non-specific abdominal pain	146 (27.2)	44 (14.9)	190 (22.8)
Biliary disease, included acute cholecystitis	66 (12.3)	47 (15.9)	113 (13.6)
Acute colonic diverticulitis	55 (10.2)	40 (13.5)	95 (11.4)
Acute appendicitis	59 (11.0)	7 (2.4)	66 (7.9)
Bowel obstruction	36 (6.7)	27 (9.1)	63 (7.6)
Ureteral stones	38 (7.1)	14 (4.7)	52 (6.2)
Constipation	15 (2.8)	22 (7.4)	37 (4.4)
Urinary, except ureteral stones	17 (3.2)	17 (5.7)	34 (4.1)
Acute pancreatitis	16 (3.0)	11 (3.7)	27 (3.2)
Gynecological disorders	14 (2.6)	7 (2.4)	21 (2.5)
Inflammatory bowel disease	15 (2.8)	3 (1.0)	18 (2.2)
Gastrointestinal cancer	4 (0.7)	13 (4.4)	17 (2.0)
Thoracic conditions, included pneumonia	11 (2.0)	3 (1.9)	14 (1.7)
Acute gastroenteritis	10 (1.9)	4 (1.4)	14 (1.7)
Incarcerated hernia	2 (0.4)	6 (2.0)	8 (1.0)
Other specific acute abdominal conditions	33 (6.1)	31 (10.5)	64 (7.7)
Total	537 (100)	296 (100)	833 (100)

Values in parentheses are percentages

Among included patients, 95 patients had a final diagnosis of acute colonic diverticulitis, including two with abscess and three with perforation and generalized peritonitis. The tentative diagnosis from the primary care physicians had been acute colonic diverticulitis in 49 patients, giving a sensitivity for this diagnosis of 52 %. The rest of the patients with a final diagnosis of acute colonic diverticulitis had been admitted from primary care physicians with the following diagnoses: NSAP (24 patients), acute appendicitis (6 patients), acute intestinal obstruction (6 patients), and other specified abdominal conditions (10 patients). In 19 patients admitted with a misdiagnosis of acute colonic diverticulitis, the final diagnosis was NSAP (7 patients), peptic ulcer (3 patients), acute appendicitis (2 patients), pancreatic cancer with metastases (1 patient), and other diagnoses in 6 patients. The proportion of patients correctly diagnosed by the primary care physicians with another diagnosis than diverticulitis was 719 out of total 738 patients, analog to a specificity of 97 %.

Table 3 shows the primary clinical scores, based on evaluation by non-specialist doctors in the emergency room, for patients with a final diagnosis of acute colonic diverticulitis. The percentages of patients with acute colonic diverticulitis increased smoothly from 1.6 % among patients with a score of zero to 100 % among those with the maximum score of 10.

**Table 3:** Primary clinical evaluation score for final diagnosis of acute colonic diverticulitis by non-specialist doctors

Non-specialist doctor’s clinical score	Diverticulitis diagnosis at discharge		Total
	No	Yes	
0	191 (98.4)	3 (1.6)	194
1	250 (98.8)	3 (1.2)	253
2	139 (97.9)	3 (2.1)	142
3	61 (89.7)	7 (10.3)	68
4	25 (78.1)	7 (21.9)	32
5	33 (78.6)	9 (21.4)	42
6	18 (62.1)	11 (37.9)	29
7	10 (45.4)	12 (54.6)	22
8	8 (26.7)	22 (73.3)	30
9	3 (20.0)	12 (80.0)	15
10	0 (0.0)	6 (100.0)	6
Total	738 (88.6)	95 (11.4)	833

Values in parentheses are percentages

*p* < 0.001 (Cochran-Armitage Trend test)

Table 4 shows the final diagnoses of all patients with clinical scores between 6 and 10. Two thirds of the patients with acute colonic diverticulitis (63/95) had high scores after the primary evaluation by the non-specialist doctors on admission.

**Table 4:** Final diagnosis in patients with acute abdominal pain and a high clinical score (6 to 10) for suspicion of acute colonic diverticulitis

Final diagnosis	Number of patients
Acute colonic diverticulitis	63
Non-specific abdominal pain	10
Constipation	4
Acute appendicitis	3
Inflammatory bowel disease	3
Urinary, except ureteral stones	3
Ureteral stones	2
Gynecological disorders	2
Peptic ulcer	2
Diverticulosis (without inflammation)	2
Biliary disease	1
Bowel obstruction	1
Acute gastroenteritis	1
Bowel perforation	1
Retroperitoneal metastases (seminoma)	1
SIRS, unknown origin	1
Osteomyelitis of left greater trochanter	1
Torsion of an epiploic appendage	1
Total	102

### The diagnostic accuracy of the initial clinical score

**Fig. 1:**
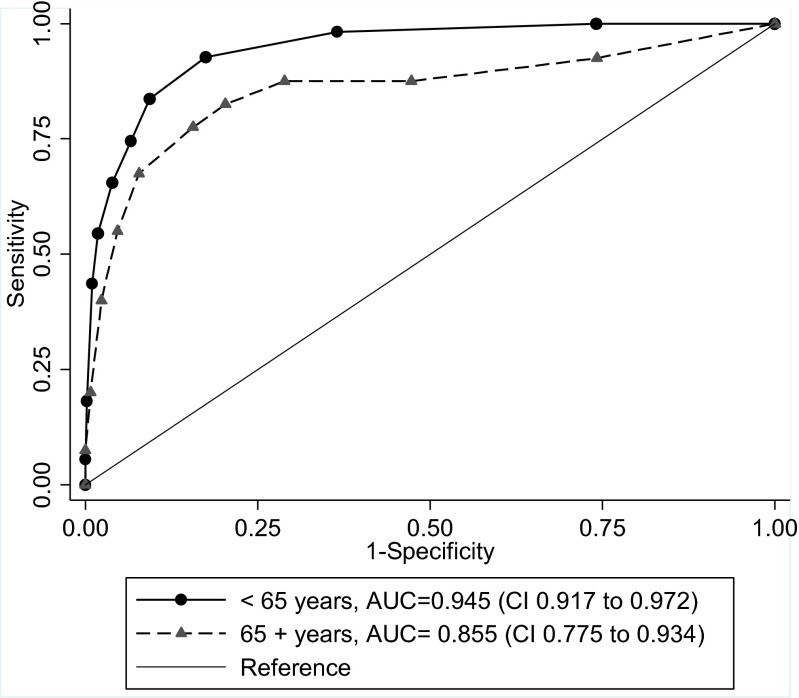
Receiver operating characteristic (ROC) curves with corresponding area under the curve (AUC) for clinical scoring of acute colonic diverticulitis in patients below and above 65 years of age admitted with acute abdominal pain

Figure 1 illustrates the ROC curve analysis used to assess the diagnostic performance of the initial clinical scores given on admission. The initial diagnostic performance ranged from excellent to outstanding discrimination, with an AUC of 0.95 (CI 0.92 to 0.97) in patients younger than 65 years and 0.86 (CI 0.78 to 0.93) in patients aged 65 years or older. The AUC was significantly higher among younger than it was among older patients (*p* = 0.036). When we chose a cutoff value of 6 on the scale from 0 to 10 in patients younger than 65 years, ROC curve analysis resulted in a sensitivity of 65 % and a specificity of 96 %. In patients 65 years and older, a cutoff value of 6 resulted in a sensitivity of 68 % and specificity of 92 %.

### The diagnostic accuracy of temperature, WBC, and CRP

**Fig. 2:**
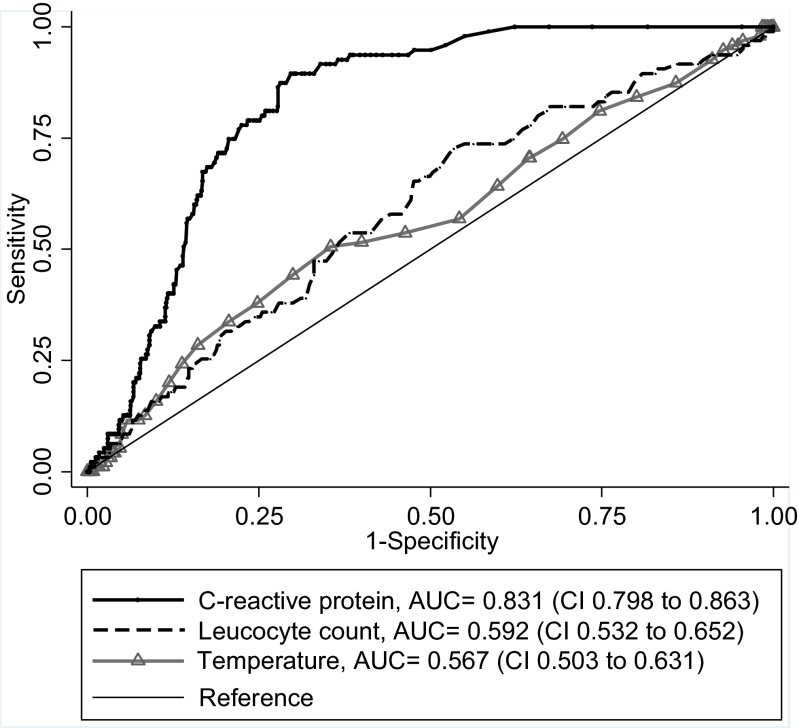
Receiver operating characteristic (ROC) curves with corresponding area under the curve (AUC) for C-reactive protein, white blood cell count, and body temperature in predicting acute colonic diverticulitis in patients admitted with acute abdominal pain

The diagnostic performance of temperature, CRP, and WBC on admission is illustrated in Fig. 2. This ROC curve analysis showed an excellent performance for C-reactive protein with an AUC of 0.83 (CI 0.80 to 0.86), while temperature and WBC had almost no discriminative power, with an AUC of 0.59 (CI 0.53 to 0.65) and 0.57 (CI 0.50 to 0.63), respectively.

## Discussion

### Main findings

This prospective series demonstrates that standard clinical evaluation, by non-specialist doctors, provides a high degree of diagnostic precision in patients with acute colonic diverticulitis. The diagnostic precision was slightly higher among patients younger than 65 years compared to those older. Furthermore, the analyses proved CRP to be the most valuable initial laboratory test in the differentiation of acute diverticulitis from other acute abdominal conditions.

### Relation to other reports

Few reports have used ROC curve analysis to assess the clinical diagnostic performance in acute colonic diverticulitis [8, 32]; a categorical scoring system similar to the one presented here has never been used. In order to compare our findings with previous studies regarding sensitivity and specificity, we chose a cutoff value of 6 on the scale from 0 to 10. The resulting sensitivities and specificities were in accordance with previous studies that reported a sensitivity of 64–71 % and a specificity of 97–98 % when evaluating clinical diagnostics in acute colonic diverticulitis [8, 9, 22–25].

The clinical diagnostic sensitivity of acute colonic diverticulitis, based on examinations before admission to hospital by the primary care physicians, was 52 %. This was inferior to the results achieved by the non-specialist doctors in the hospital, but comparable to a previous study on diagnostic sensitivity of acute colonic diverticulitis in primary health care [24]. Various factors have impact on this finding: increased focus on patients with acute colonic diverticulitis, because of the study and the selection of patients admitted to a surgical department, advantage of the tentative diagnosis from the primary care physicians, and access to previous hospital records, body temperature, and blood tests in all included cases.

In the present study, the accuracy of the clinical diagnosis was slightly better in younger patients. The presentation of acute abdominal pain among elderly may be different from that seen in younger age groups. Elderly patients tend to have more vague and non-specific symptoms, broader alternatives of differential diagnosis, altered clinical signs that do not correlate with disease severity, higher incidence of comorbidity and multi-pharmacy, and communication difficulties because of hearing and cognitive impairment [26, 27]. This makes elderly patients more prone to misdiagnosis than younger patients [28].

ROC curve analysis to assess CRP in the diagnostics of acute colonic diverticulitis has previously been reported with an AUC ranging from 0.72 to 0.94 [8, 30–34], which is comparable to the present results. Furthermore, these studies confirm WBC count and body temperature to be without discriminative power in distinguishing diverticulitis from other patients with acute abdominal pain, with an AUC between 0.54 and 0.57 [8, 33].

Most previous studies have focused on these parameters in the differentiation between uncomplicated and complicated acute colonic diverticulitis [29, 32, 33]; also demonstrating that CRP, other than WBC count, was the most important biochemical marker.

### Practical implication

This study confirms that a clinical diagnosis of acute colonic diverticulitis is achievable by non-specialist doctors at the emergency department and applies specifically to patients younger than 65 years with localized tenderness in the left lower quadrant and an elevated CRP.

Previous studies report other criteria as significant in the selection of patients suitable for out-patient diagnosis and treatment such as a CRP cutoff value between 150 and 200 mg/L, absence of vomiting, significant fever and signs of generalized peritonitis, absence of compromised immune status, and significant comorbidities. Of additional importance is a close follow-up and the possibility of a secondary evaluation if symptoms worsen [8, 31–37]. The diagnosis would be even more strengthened in patients who have recurrent symptoms, with colonic diverticular disease verified on a pervious CT scan.

Out-patient treatment would include oral analgesics, with or without oral antibiotics. Follow-up, including clinical examination and CRP with the possibility of ambulant CT scan on day 4 and referral to follow-up endoscopy or CT colonography after 6 weeks of improvement, has been suggested [38].

This evidence should form the basis for a clinical care pathway. A structural approach, involving both the primary and secondary healthcare system, would increase the quality of treatment, define an appropriate level of treatment for the individual patient, and reduce the increasing rates of admission to hospital among patients with acute colonic diverticulitis.

### Weakness of the study

Not all patients admitted to hospital because of acute abdominal pain in the study period were included in this study. There were no statistical differences in gender, age, subtype of acute colonic diverticulitis, or hospital stay when comparing patients included or not, as shown in Table 1. The included patients seem to be a representative selection of all patients admitted with acute abdominal pain, although a more complete inclusion would have increased the precision of the findings.

Another limitation is the probable selection bias based on the hospital doctors’ awareness of the study. However, the percentage of patients with acute colonic diverticulitis in relation to other types of acute abdomen conditions generally matches other studies [39].

### Strengths of the study

The present prospective study is the first to use a fine graded categorized clinical score and subsequent ROC curve analysis in the evaluation of clinical diagnostic accuracy in patients with acute colonic diverticulitis. The advantage of ROC curve analysis in the present study was the possibility to consider the complete spectrum of the observed results, not only the mean or dichotomous variable denoting “yes or no.”

The study highlights that non-specialist doctors, usually in their first year of a clinical career, were able to clinically diagnose acute colonic diverticulitis in patients with acute abdominal pain with a high degree of accuracy. This reflects the possibility of a similar standardized approach applied in the out-patient setting, reducing the need for further referral to hospitals, especially in cases of suspected uncomplicated acute colonic diverticulitis.

## Conclusion

This prospective study demonstrates that standard clinical evaluation by non-specialist doctors based on history, physical examination, and initial blood tests on admission provides a high degree of diagnostic precision in patients with acute colonic diverticulitis.

Furthermore, the analyses proved CRP to be a valuable initial laboratory test in the differentiation of acute colonic diverticulitis from other acute abdominal conditions.
